# Subcutaneous immunoglobulin in primary immunodeficiency – impact of training and infusion characteristics on patient-reported outcomes

**DOI:** 10.1186/s12865-020-00371-y

**Published:** 2020-08-10

**Authors:** R. Mallick, T. Henderson, B. J. Lahue, A. Kafal, P. Bassett, C. Scalchunes

**Affiliations:** 1 CSL Behring, King of Prussia, PA USA; 2 Immune Deficiency Foundation, Towson, MD USA; 3 Alkemi LLC, Stratton, VT USA; 4 Meridian HealthComms Ltd, Manchester, UK

**Keywords:** Primary immunodeficiency, Subcutaneous immunoglobulin (SCIG), Patient reported outcomes, Treatment satisfaction questionnaire for medication (TSQM), PROMIS fatigue, Infusion parameters

## Abstract

**Background:**

Subcutaneous immunoglobulin (SCIG) is increasingly utilized in primary immunodeficiency (PI). Understanding factors associated with treatment experience and satisfaction can optimize patient outcomes. We analyzed Immune Deficiency Foundation (IDF) survey data to evaluate patient-reported outcomes (PROs) in relation to SCIG training and infusion characteristics. Respondents’ PRO scores were rank ordered into ‘best’, ‘intermediate’, and ‘worst’ tertiles. Predicted probabilities of being in the best tertile with any combination of characteristics were generated for each PRO.

**Results:**

In 366 SCIG respondents, higher odds of being in the best PRO tertile were driven by favorable training characteristics (particularly, higher confidence post-training and no training barriers) and efficient infusions (infusion preparation ≤20 min and actual infusion < 2 h). Age (≤17 years old) and treatment experience (> 2 years) increased the odds of being in the best tertiles. Compared with the least favorable training/infusion characteristics, those with the most favorable training/infusion characteristics had higher predicted probabilities of being in the best tertiles: TSQM *side effects*, 59% vs 4%; *convenience*, 52% vs 4%; *effectiveness*, 27% vs 13%; *global*, 26% vs 3%; PROMIS Fatigue, 44% vs 18%.

**Conclusions:**

Increased experience with SCIG consistently improved PROs, but our findings predicted that enhanced training and infusion characteristics improve patient treatment satisfaction beyond that achieved by experience alone.

## Background

Primary immunodeficiencies (PIs) are a heterogeneous group of > 350 genetic disorders, characterized by an increased susceptibility to infections as a result of impaired immune system function [1, 2]. In addition to persistent and recurrent infections, patients can experience complications including allergies, malignancies, and autoimmune diseases [3]. In the United States (US), PI has an estimated prevalence of 1 in 1200 people [4]. As a chronic disease, PIs and specifically primary antibody deficiencies, usually require lifelong immunoglobulin (Ig) G replacement therapy, administered as either intravenous IgG (IVIG) or subcutaneous IgG (SCIG).

Quality of life (QOL) assessments typically focus on disease-related aspects of patient well-being but can also reflect the impact of treatment on patients [5, 6]. Chronic diseases place a long-term burden on general satisfaction, physical function, emotional well-being, work productivity, and family life [7]. Regular long-term treatment regimens interfere with daily life and can act as constant reminders of disease [8]. Arguably, the associated treatment burden increases with treatment complexity and duration; endeavors to make long-term treatments simpler, easier, and more convenient can have a favorable effect on patients [5].

In PI, where oral treatment is not an option, many patients prefer the possibility of flexible subcutaneous dosing at home and no longer needing to travel to a healthcare facility to obtain IVIG [9, 10]. Studies have shown that for many patients, QOL and self-perception of their health is improved following a transition from IVIG to SCIG [9, 11–13]. SCIG offers the convenience of self-administration, eliminates the need for venous access, enables shorter infusions with smaller volumes, and yields an improved safety profile [10, 14, 15]. Due to the reduced treatment burden, SCIG is a popular alternative to IVIG for many patients with PI [13].

Transition to a self-administration route is usually associated with patients developing a better overall knowledge of their disease over time [15]. Evidence suggests that patients with more incentives to self-administer (e.g. those who travel frequently or are employed) are more likely to want to transition to SCIG and may be more engaged in learning self-administration and derive greater perceived benefit from efficient infusions [16]. Yet, some patients may be discouraged from switching from IVIG to SCIG on account of perceived inconvenience, concerns about adverse effects at home, and fear of needle sticks [13]. A systematic understanding of what factors drive efficiency in infusion and learning, and in turn treatment satisfaction and well-being, can help guide best practices.

The Immune Deficiency Foundation (IDF) conducts regular surveys exploring PI in order to provide evidence on patient health and well-being. We report on an IDF survey that focused in-depth on key patient-reported outcomes (PROs) — treatment satisfaction, fatigue, and general health perception (GHP) — in relation to SCIG administration and training experiences. In particular, we identified predictors associated with higher GHP or treatment satisfaction, less fatigue, and favorable infusion and training experiences in a cohort of patients on SCIG. Based on these findings, we provide evidence-based recommendations for improving outcomes in patients with PIs.

## Results

**Fig. 1:**
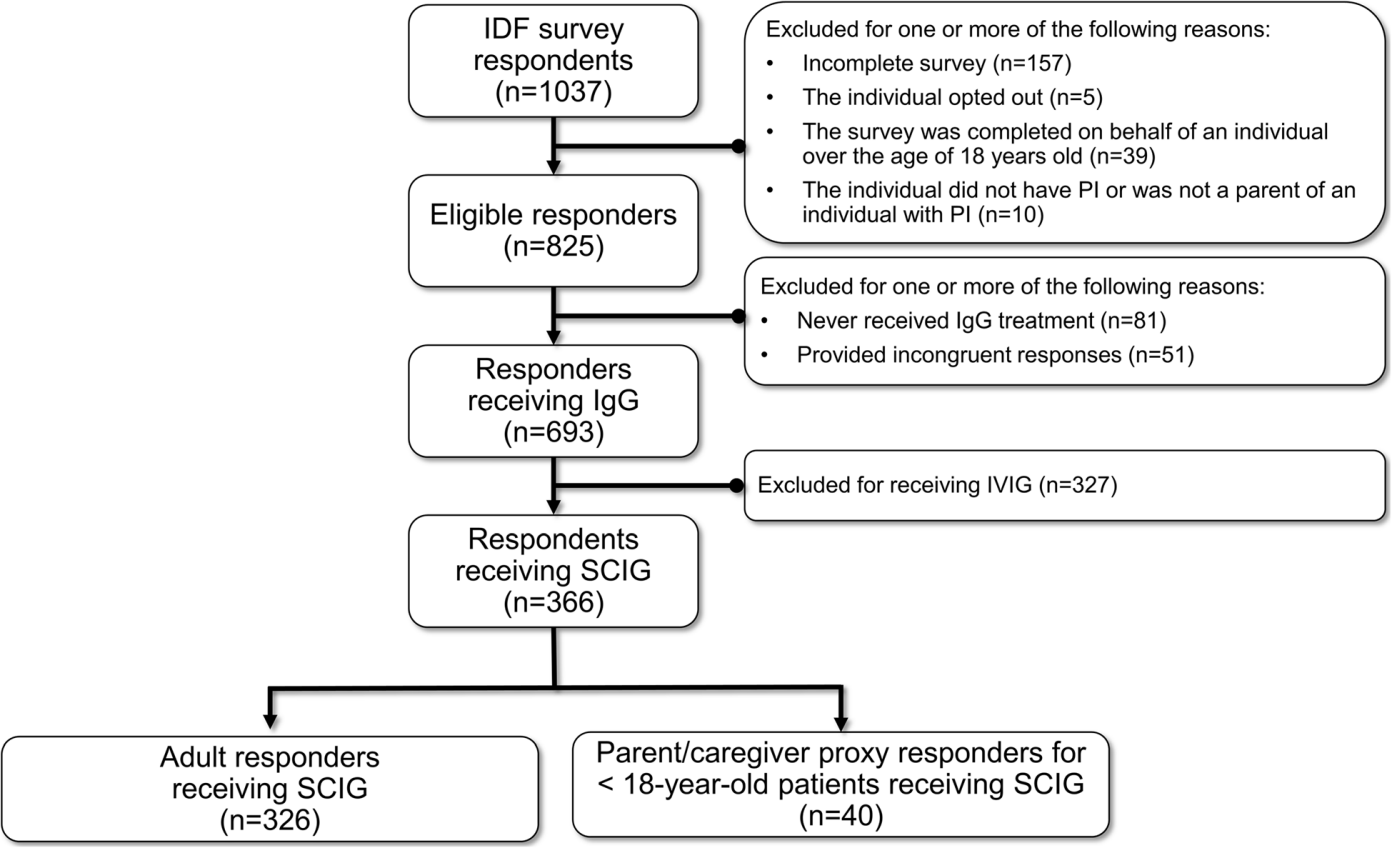
Criteria used to include responders in the study. IDF, Immune Deficiency Foundation; IgG, immunoglobulin G; IVIG, intravenous immunoglobulin; PI, primary immunodeficiency; SCIG, subcutaneous immunoglobulin

In total, 35.3% (366/1037) of responders to the IDF survey were included in our analyses (see Fig. 1 for survey respondent flow diagram). Patients were excluded for one or more of the following reasons: an incomplete survey; the individual opted out; the survey was completed on behalf of an individual over the age of 18; the individual did not have PI or was not a parent of an individual with PI; had never received IgG treatment; provided incongruent response; receiving IVIG. Of the eligible responders receiving SCIG, adult patients accounted for 89% (*n* = 326) with the remaining being parent/caregiver proxy responses for < 18-year-old patients (*n* = 40) (Table 1). Table 2 provides the tertile thresholds for ranking each PRO based on respondent scoring.

**Table 1:** Respondent demographics

Characteristics		Respondent, N (%)
**Non-infusion characteristics**		
Age	0–17 years	40 (11%)
	≥18 years	326 (89%)
Experience with IgG prior to SCIG	Ig-naïve	138 (38%)
	Ig-experienced	226 (62%)
Experience with SCIG	< 2 years	129 (35%)
	≥2 years	235 (65%)
SCIG transition decision driver	Prescriber	102 (47%)
	Patient	96 (45%)
	Other	17 (8%)
SCIG treatment decision driver	Prescriber	238 (65%)
	Patient	95 (26%)
	Other	33 (9%)
Time on treatment	< 2 years	76 (21%)
	≥2 years	290 (79%)
**SCIG training characteristics**		
Training location	Hospital	11 (3%)
	Doctor’s office	55 (15%)
	Home	274 (75%)
	Other	23 (6%)
Who conducted training	Clinical staff	43 (12%)
	Specialty pharmacy	278 (78%)
	Nurse	30 (8%)
	Other	6 (2%)
Number of training sessions required	1–3	291 (80%)
	≥4	71 (20%)
Length of training sessions	≤2 h	225 (63%)
	> 2 h	131 (37%)
Competence of trainer^a^	1–5	72 (20%)
	6–7	290 (80%)
Knowledge of trainer^a^	1–5	99 (28%)
	6–7	261 (72%)
Ease of training^b^	1–5	103 (28%)
	6–7	263 (72%)
Confidence after training^a^	1–5	110 (30%)
	6–7	251 (70%)
Satisfaction with quality of training^a^	1–5	76 (21%)
	6–7	286 (79%)
Barriers to training	No	245 (82%)
	Yes	53 (18%)
**SCIG infusion parameters**		
Preparation duration	≤20 mins	196 (54%)
	> 20 mins	168 (46%)
	Median [IQR] mins	20 [15, 44]
Actual infusion time	< 2 h	161 (44%)
	≥2 h	203 (56%)
	Median [IQR] mins	120 [80, 150]
Complete infusion time (inc. prep and clean-up)	≤3 h	237 (65%)
	> 3 h	127 (35%)
	Median [IQR] mins	155 [115, 209]
Frequency of infusions^c^	> 1 per week	28 (8%)
	Weekly or up to every 2 weeks	284 (79%)
	Every 3 or 4 weeks	48 (13%)
Number of sites per infusion	1–3	212 (58%)
	≥4	152 (42%)
Product concentration	10%	118 (33%)
	20%	243 (67%)

*IQR* interquartile range, *IgG* immunoglobulin G, *SCIG* subcutaneous immunoglobulin

^a^On an anchored numeric scale from 1 to 7 (1 = not very competent/knowledgeable/confident/satisfied and 7 = very competent/knowledgeable/confident/satisfied) ^b^On an anchored numeric scale from 1 to 7 (1 = very difficult and 7 = very easy)

^c^Patients responding ‘other’ omitted from summary as frequency unknown

**Table 2:** General health perception, treatment satisfaction, and fatigue

Characteristics		Range
**General health perception**		
	T1 - best	≥6
	T2 - intermediate	5
	T3 - worst	≤4
	Mean ± SD	4.6 ± 1.3
**TSQM scores**		
Total	T1 - best	≥81
	T2 - intermediate	68–80
	T3 - worst	≤67
	Mean ± SD	74 ± 16
*Effectiveness*	T1 - best	≥76
	T2 - intermediate	61–75
	T3 - worst	≤60
	Mean ± SD	71 ± 21
*Side effects*	T1 - best	100
	T2 - intermediate	70–99
	T3 - worst	≤69
	Mean ± SD	81 ± 26
*Convenience*	T1 - best	≥81
	T2 - intermediate	66–80
	T3 - worst	≤65
	Mean ± SD	70 ± 17
*Global*	T1 - best	≥86
	T2 - intermediate	71–85
	T3 - worst	≤70
	Mean ± SD	78 ± 19
**PROMIS fatigue T-scores**		
	T1 - best	≤53
	T2 - intermediate	54–60
	T3 - worst	≥61
	Mean ± SD	57 ± 9

Respondents were ranked by their scores and divided into tertiles (T1, T2 and T3) corresponding to best, intermediate, and worst, respectively. GHP was measured on a 1–7 scale (1 = poor health and 7 = excellent health). TSQM transformed scores were measured on a 0–100 scale (0 = worst satisfaction and 100 = perfect satisfaction). Fatigue was assessed using the PROMIS Fatigue Short Form 7a and Parent/Caregiver Proxy respectively ( 29.4 = least fatigue and 83.2 = most fatigue)

*GHP* General health perception, *PROMIS* Patient-Reported Outcome Management Information System, *SD* standard deviation, *SCIG* subcutaneous immunoglobulin, *TSQM* Treatment Satisfaction Questionnaire for Medication

### Predictors of GHP

Respondents were > 8 times more likely to be in the best GHP tertile if they were in the best tertile for Patient-Reported Outcome Management Information System (PROMIS) Fatigue (*p* < 0.001). Additionally, those with > 2 years’ IgG experience prior to SCIG (odds ratio [OR] = 2.36) (*p* = 0.01), higher confidence after training (OR = 2.18) (*p* = 0.03), and best tertile Treatment Satisfaction Questionnaire for Medication (TSQM) *effectiveness* scores (OR = 2.73) (*p* = 0.001) had higher odds of being in the best tertile for GHP. Full results are in Table 3.

**Table 3:** Predictors for being in the best tertile of GHP scores

Predictor	Category	Logistic regression			Linear regression		
		OR	95% CI	***p***-value	Coefficient	95% CI	***p***-value
Age	0–17 years	1		0.006	0		0.004
	≥18 years	0.30	0.12, 0.70		−0.52	−0.88, −0.16	
Experience with IgG prior to SCIG	Ig-naïve	1		0.01			
	Ig-experienced	2.36	1.22, 4.54				
Time on treatment	< 2 years				0		0.06
	≥2 years				0.27	−0.01, −0.54	
Confidence after training^a^	1–5	1		0.03			
	6–7	2.18	1.07, 4.44				
Number of sites	1–3	1		0.01	0		0.04
	≥4	0.44	0.24, 0.84		−0.24	−0.47, −0.02	
TSQM *effectiveness* score^b^	T2 + T3, ≤75	1		0.001	0.13	0.07, 0.18	< 0.001
	T1, ≥76 (best)	2.73	1.50, 4.80				
PROMIS Fatigue^c^	T2 + T3, ≥54	1		< 0.001	−0.33	−0.39, −0.26	< 0.001
	T1, ≤53 (best)	8.26	4.56, 15.0				

Multivariate logistic regression and linear regression models calculated predictors for being in the best tertile of GHP scores. GHP was measured on an anchored numeric 1–7 scale (1 = poor health and 7 = excellent health), where respondents were grouped in T2 + T3 (intermediate/worst) if they had a score of ≤ 5 and in T1 (best) if they scored 6 or 7. PROMIS Fatigue T-scores are obtained from published raw score to T-score concordance tables of the PROMIS Fatigue Short Form 7a. With 5 levels on each of the 7 items, the raw scores vary from 7 to 35 and are converted to corresponding T-scores in the range of 29.4 (least fatigue) to 83.2 (most fatigue). TSQM transformed scores (T-scores) were measured on a 0–100 scale (0 = worst satisfaction and 100 = perfect satisfaction)

*CI* confidence interval, *GHP* general health perception, *IgG* immunoglobulin G, *OR* odds ratio, *PROMIS* Patient-Reported Outcome Management Information System, *SCIG* subcutaneous immunoglobulin, *SD* standard deviation, *TSQM* Treatment Satisfaction Questionnaire for Medication

^a^Predictor on an anchored numeric scale from 1 to 7 (1 = not very confident and 7 = very confident). The logistic regression yields an OR which predicts the likelihood of each category achieving the desired best tertile, and a significant OR > 1 implies higher odds than with the reference category. The least squares regression models score on a continuous linear scale using the original 1–7 scale, where a higher coefficient implies a higher GHP

^b^Regression coefficient reported for a 0.5 SD increase in score (equivalent to 10 units)

^c^Regression coefficient reported for a 0.5 SD increase in score (equivalent to 5 units). The model had an R^2^ = 36.2%, suggesting that over a third of scores can be explained by the factors in the final model

### Predictors of TSQM and PROMIS Fatigue: role of favorable training characteristics

Favorable training characteristics translated to higher odds of being in the best tertile for TSQM domains. For *effectiveness:* absence of training barriers was associated with higher odds of being in the best tertile (*p* = 0.01). In the corresponding continuous scale (linear) model, requiring fewer training sessions was significantly associated with a higher *effectiveness* score (*p* = 0.01). For *side effects:* a higher confidence after training was associated with higher odds of being in the best tertile (*p* = 0.004). The linear model revealed that fewer training sessions translated to a better s*ide effect* score (*p* = 0.05). For *convenience:* higher confidence after training and absence of training barriers were associated with higher odds of being in the best tertile (*p* = 0.001 and *p* = 0.04, respectively). In the linear model, respondents rating training as easier were more likely to have a higher *convenience* score. For *global:* more competent trainers were associated with better odds of a higher *global* score (*p* = 0.001), and in the linear model, high confidence after training was significantly associated with high *global* scores (*p* = 0.002). In addition, training sessions that were > 2 h resulted in better odds of a higher *global* score. A higher confidence after training was associated with reduced fatigue (*p* = 0.01) (Table 4).

**Table 4:** Predictors for being in the best tertile of PROMIS Fatigue T-scores

Predictor	Category	Logistic regression			Linear regression		
		OR	95% CI	***p***-value	Coefficient	95% CI	***p***-value
Confidence after training^a^	1–5	1		0.01	0		0.02
	6–7	1.95	1.16, 3.28		−0.24	−4.5, −0.4	
Actual infusion time	≥2 h	1		0.01	0		0.06
	< 2 h	1.80	1.14, 2.82		−1.8	−3.7, −0.1	

Fatigue – multivariate logistic regression and linear regression models calculated predictors for being in the best tertile of PROMIS Fatigue T-scores. PROMIS Fatigue scores were transformed to a 1–100 scale (0 = least fatigue, 100 = most fatigue), where respondents were grouped in T2 + T3 (intermediate/worst) if they had a fatigue score > 53 and in T1 (best) if they had a fatigue score ≤ 53

*CI* confidence interval, *OR* odds ratio, *PROMIS* Patient-Reported Outcome Management Information System

^a^Predictor on an anchored numeric scale from 1 to 7 (1 = not very confident and 7 = very confident). The logistic regression provides an OR which predicts the likelihood of that category falling into T1, where the higher number corresponds to better odds. The least squares regression considers scores on a continuous scale using the original 0–100 scale, where a lower coefficient implies a better fatigue score for that category. The least squares  model had an R^2^ = 2.5%, suggesting that factors examined were not strongly associated with a respondent’s PROMIS Fatigue score

### Predictors of TSQM and PROMIS Fatigue: role of efficient infusions

Efficient infusions increased the odds of high (best tertile) TSQM scores for most domains (Table 5). A shorter infusion preparation duration resulted in better odds of being in the best tertile for *convenience* (*p* < 0.001) and *global* (*p* = 0.03) domains. There was also an association between shorter infusion preparation, actual infusion times, and being in the best tertile for *side effects* (*p* = 0.007 and 0.03, respectively). Finally, having an actual infusion time ≤ 2 h was also associated with reduced fatigue (*p* = 0.01) (Table 4).

**Table 5:** Predictors for being in the best tertile of TSQM total and domain scores

Predictor	Category	Logistic regression			Linear regression		
		OR	95% CI	***p***-value	Coefficient	95% CI	***p***-value
***Total***							
Age	0–17 years	1		0.08	0		0.005
	≥18 years	0.51	0.23, 1.09		−7.3	−12.3, −2.2	
Time on treatment	≤2 years	1		0.008	0		0.03
	> 2 years	2.07	1.21, 3.52		4.0	0.5, 7.5	
Ease of training^a^	1–5				0		0.05
	6–7				4.2	0.0, 8.4	
Confidence after training^a^	1–5	1		0.03	0		0.001
	6–7	1.89	1.08, 3.33		6.6	2.7, 10.4	
Barriers to training	Yes	1		0.06	0		0.002
	No	1.96	0.96, 4.00		6.6	2.4, 10.8	
Infusion preparation duration	> 20 mins	1		0.08	n/s	n/s	n/s
	≤20 mins	1.59	0.95, 2.65				
***Effectiveness***							
Time on treatment	≤2 years	1		0.004	0		0.05
	> 2 years	2.56	1.35, 4.88		5.7	−0.1, 11.4	
Training sessions required	1–3				0		0.01
	≥4				−7.5	−13.1, −1.8	
Barriers to training	Yes	1		0.01	0		0.005
	No	2.43	1.23, 4.81		8.5	2.6, 14.4	
***Side effects***							
Age	0–17 years	1		0.04	0		0.007
	≥18 years	0.45	0.21, 0.96		−10.7	−18.4, −2.9	
Time on treatment	≤2 years	1		0.003	0		0.03
	> 2 years	2.36	1.34, 4.16		7.1	0.7, 13.4	
Training sessions required	1–3				0		0.05
	≥4				−6.8	−13.5, −0.1	
Confidence after training^a^	1–5	1		0.004	0		< 0.001
	6–7	2.04	1.25, 3.32		11.1	5.7, 16.4	
Infusion preparation duration	> 20 mins	1		0.007	0		0.002
	≤20 mins	1.88	1.19, 2.98		8.1	3.1, 13.1	
Actual infusion time	> 2 h	1		0.03	n/s	n/s	n/s
	≤2 h	1.70	1.06, 2.73				
Frequency of infusions	> 1 per week	1		0.008	0		0.003
	Weekly/up to every 2 weeks	4.09	1.66, 10.10		14.5	5.3, 23.7	
	Every 3 or 4 weeks	4.68	1.54, 14.20		19.5	8.1, 30.9	
***Convenience***							
Age	0–17 years	1		0.03	0		0.01
	≥18 years	0.41	0.19, 0.92		−7.4	−13.1, −1.7	
Experience on SCIG	≤2 years	1		0.04			
	> 2 years	1.80	1.02, 3.12				
Ease of training^a^	1–5				0		0.003
	6–7				7	2.5, 11.6	
Confidence after training^a^	1–5	1		0.001	0		< 0.001
	6–7	3.00	1.59, 5.65		7.9	3.6, 12.2	
Barriers to training	Yes	1		0.04	0		0.006
	No	2.23	1.02, 4.88		6.6	1.9, 11.4	
Infusion preparation duration	> 20 mins	1		< 0.001	0		0.05
	≤20 mins	1.95	1.13, 3.35		3.8	0.0, 7.5	
***Global***							
Age	0–17 years	1		0.04	0		0.007
	≥18 years	0.46	0.22, 0.96		−8.0	−13.8, −2.2	
Time on treatment	≤2 years	1		0.001	0		< 0.001
	> 2 years	3.39	1.66, 6.93		10.7	6.1, 15.3	
Competence of trainer^a^	1–5	1		0.001			
	6–7	3.47	1.66, 7.22				
Confidence after training^a^	1–5				0		0.002
	6–7				6.4	2.5, 10.4	
Length of training session	≤2 h	1		0.006	0		0.01
	> 2 h	2.02	1.22, 3.35		4.8	1.0, 8.7	
Infusion preparation duration	> 20 mins	1		0.03	0		0.001
	≤20 mins	1.76	1.07, 2.97		6.2	2.5, 9.8	

Treatment satisfaction – multivariate logistic regression and linear regression models calculated predictors for being in the best tertile of TSQM total and domain scores. TSQM scores were transformed to a 1–100 scale (1 = worst satisfaction and 100 = perfect satisfaction), where respondents were grouped in T2 + T3 (intermediate/worst) if they had a lower score and in T1 (best) if they had a higher score (see Table 2 for exact tertile ranges)

*CI* confidence interval, *n/s* not significant, *OR* odds ratio, *SCIG* subcutaneous immunoglobulin, *TSQM* Treatment Satisfaction Questionnaire for Medication

^a^Predictor on an anchored numeric scale from 1 to 7 (1 = very difficult or not very confident/competent and 7 = very easy or very confident/competent). The logistic regression provides an OR which predicts the likelihood of that category falling into T1, where the higher number corresponds to better odds. The least squares regression considers scores on a continuous scale using the 0–100 scale, where a higher coefficient implies a higher TSQM score

### Predictors of TSQM and PROMIS Fatigue: role of respondent experience and demographics

Those with ≥2 years on SCIG treatment were significantly more likely to be in the best tertile of total TSQM (*p* = 0.008) and *global* (*p* = 0.001). Also, younger patients were more likely to be in the best tertile of *global* (*p* = 0.04) (Table 5).

### Predictors of favorable infusion and training

Finally, we evaluated predictors of efficient training and efficient infusions. Shorter infusion and preparation duration were more likely with training conducted in the doctor’s office rather than at home (*p* = 0.006). The odds of completing an infusion in ≤2 h were 60% higher with a 20% concentration product, relative to a 10% concentration (Table 6). A high ease of training was also associated with a 15% shorter infusion time. Respondents reporting easier training had preparation duration up to 30% shorter. Respondents reporting no barriers to training were also more likely to have shorter preparation duration.

**Table 6:** SCIG infusion parameters

Predictor	Category	Logistic regression			Linear regression		
		OR	95% CI	***p***-value	Ratio	95% CI	***p***-value
**Infusion time (actual)**	*Probability of ≤2 h* *infusion time (*vs *ref group [> 2 h])*						
Age (years)	0–17	1.93	0.97, 3.84	0.06	0.78	0.63, 0.96	0.02
	≥18 (ref group)	1			1		
Training location	Home (ref group)	1		0.006			
	Hospital	1.03	0.30, 3.57				
	Doctor’s office	3.05	1.63, 5.70				
	Other	0.95	0.39, 3.30				
Who conducted training	Clinical staff (ref group)				1		< 0.001
	Specialty pharmacy				1.54	1.26, 1.90	
	Nurse				1.49	1.11, 2.00	
Ease of training^a^	1–5 (ref group)				1		0.03
	6–7				0.84	0.73, 0.98	
Product concentration	10% (ref group)	1		0.05			
	20%	1.6	1.00, 2.56				
**Infusion time (preparation)**	*Probability of ≤20 mins* *preparation time (*vs *ref group [> 20 mins])*						
Ease of training^a^	1–5 (ref group)	1		0.001	1		0.008
	6–7	2.41	1.41, 4.14		0.7	0.54, 0.91	
Barriers to training	Yes (ref group)	1		0.003	1		0.001
	No	2.63	1.40, 4.93		0.62	0.46, 0.85	
SCIG transition decision driver	Prescriber				1		0.05
	Patient				1.34	1.05, 1.71	
	Other				1.25	0.83, 1.88	
**Total SCIG training time**	*Probability of ≤4 h* *training time (*vs *ref group [> 4 h])*						
Age (years)	0–17	2.23	1.05, 4.72	0.4			
	≥18 (ref group)	1					
Training location	Hospital (ref group)				1		0.001
	Doctor’s office				1.43	0.90, 2.26	
	Home				1.92	1.26, 2.95	
	Other				1.58	0.94, 2.65	
Knowledge of trainer^a^	1–5 (ref group)	1		0.006	1		0.002
	6–7	2.02	1.22, 3.35		1.3	1.10, 1.54	
Ease of training^a^	1–5 (ref group)	1		< 0.001	1		< 0.001
	6–7	2.44	1.50, 3.98		0.65	0.55, 0.77	

Multivariate logistic regression and linear regression models of predictors for more efficient infusions and training

*CI* confidence interval, *OR* odds ratio, *SCIG* subcutaneous immunoglobulin

^a^Predictor on an anchored numeric scale from 1 to 7 (1 = very difficult or not very knowledgeable and 7 = very easy or very knowledgeable). ORs expressed as the probability relative to the probability in a reference group (ref group). For the least squares regression, the analysis was performed using multiple linear regression with all outcomes analyzed on the log scale. Due to this log transformation, the size of the relationship between each factor and the outcome is given in the form of ratios to give the relative difference in time in for each predictor relative to the reference group

The total time requirement for SCIG training (number of training sessions multiplied by length of sessions) was analyzed as the dependent variable (Table 6). A shorter total training time was predicted by a greater ease of training; over 60% of respondents reporting very easy training completed their training in ≤4 h. The linear models indicated that respondents trained at hospital were more likely to complete this in a total ≤4 h compared with those trained at home or in the doctor’s office (*p* = 0.001). However, trainers perceived to be more knowledgeable were associated with longer overall training times (*p* = 0.002).

### Associations between training variables

Ease of training was found to be significantly, positively associated with the knowledge of the trainer (correlation coefficient [CC] = 0.13, *p* = 0.02) and significantly, negatively with the total training time (Spearman’s CC = −0.30, *p* = < 0.001) variables. The association between the knowledge of the trainer and the total training time was not significant (Spearman’s CC = 0.09, *p* = 0.09).

Ease of training and total training time variables varied significantly (*p* = 0.01 and *p* = < 0.001, respectively) between the four training locations (home, hospital, doctor’s office, or other) (Table 7). Overall, the median rating (interquartile range [IQR]) for ease of training was higher for respondents trained in hospital or a doctor’s office (7 [6, 7] for both locations), compared with respondents trained at home (6 [5, 7]) or in other locations (6 [4, 7]). The total training time was lowest in those trained in hospital, with a median time of 1 h, whereas in all other locations (home, doctor’s office, and other), the median time was 4 h. No significant difference was found between the knowledge of trainer and the different training locations (*p* = 0.21).

**Table 7:** The effect of different training locations upon ease of training, total SCIG training time, and knowledge of trainer

Variable	Home	Hospital	Doctor's Office	Other	***p***-value
**Ease of training**^**a**^	6 [5, 7]	7 [6, 7]	7 [6, 7]	6 [4, 7]	0.01
**Total SCIG training time (hours)**	4 [2, 8]	1 [0.5, 2]	4 [1, 6]	4 [2, 6]	< 0.001
**Knowledge of trainer**^**b**^	7 [5, 7]	6 [4, 7]	7 [6, 7]	7 [5, 7]	0.21

Results are stated as median (IQR) for each training variable. The association between training location and other training variables was calculated using a Kruskal-Wallis test

*IQR* interquartile range, *SCIG* subcutaneous immunoglobulin

^a^The ease of training variable was measured on a scale from 1 to 7 (1 = very difficult and 7 = very easy) ^b^The knowledge of trainer variable was measured on a scale from 1 to 7 (1 = not very knowledgeable and 7 = very knowledgeable)

### Projected impact of favorable training/infusion characteristics on achieving best PRO tertiles

**Fig. 2:**
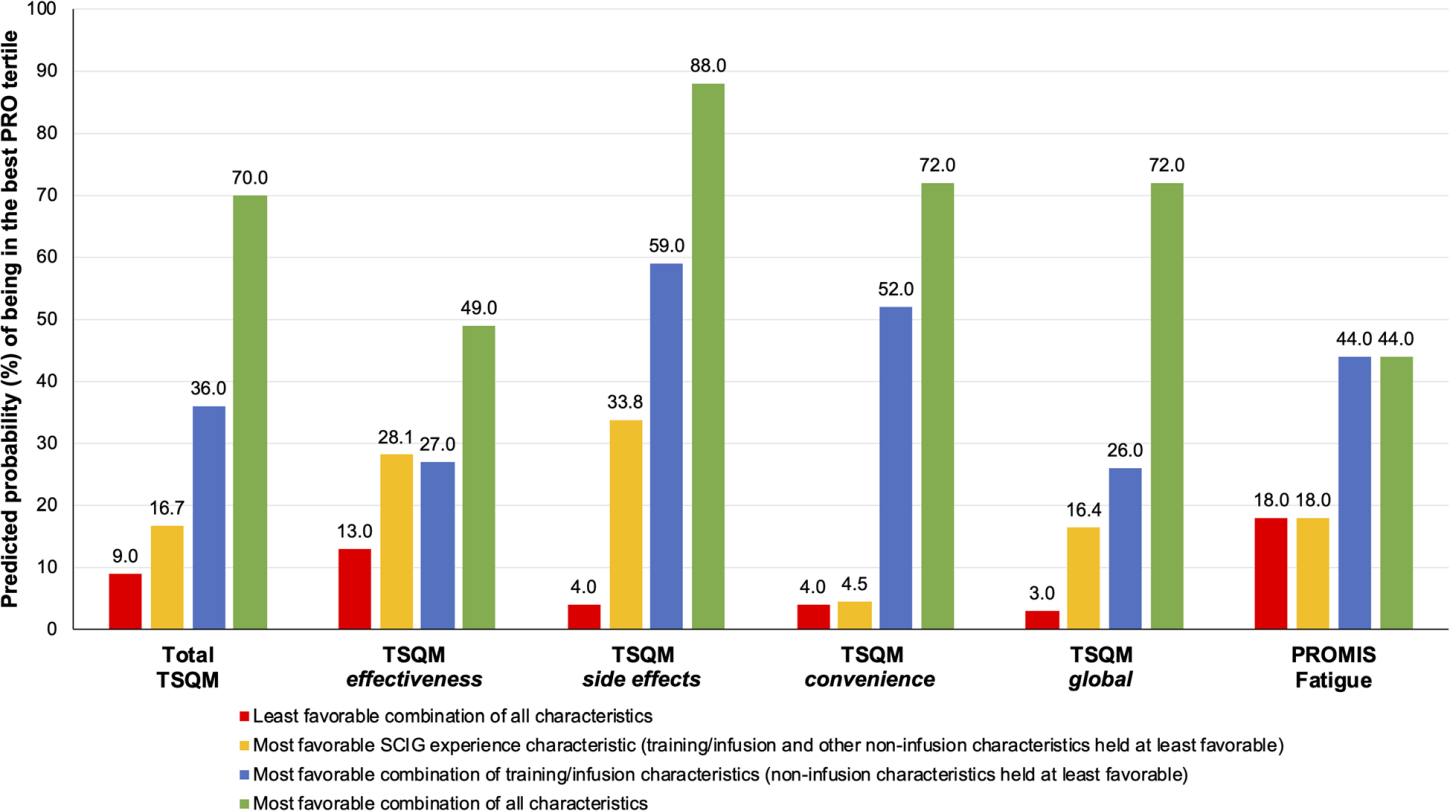
Predicted probability (%) of being in the ‘best’ PRO tertile. This measures the impact of varying all characteristics, training/infusion characteristics alone, or SCIG experience alone. Red and green bars represent the overall theoretical best and worst percent probability of being in the ‘best’ PRO tertile when all characteristics (see Table 1 for full list) were allowed to vary. PRO, patient-reported outcome; PROMIS, Patient-Reported Outcome Management Information System; SCIG, subcutaneous immunoglobulin; TSQM, Treatment Satisfaction Questionnaire for Medication

Estimated logit link functions from the PRO models were exponentiated to yield predicted probabilities of being in the best PRO tertiles, at various levels of identified predictors. Figure 2 presents the predicted probabilities of achieving the best TSQM domain tertiles, when we (i) varied all characteristics from the least to most favorable, (ii) varied only training and infusion characteristics from least to most favorable (non-infusion characteristics held at least favorable), and (iii) varied only SCIG experience (training/infusion and other non-infusion characteristics held at least favorable).

Predicted probabilities of achieving the best tertile when all patient characteristics were varied from least to most favorable ranged from: 4 to 88% *(side effects),* 3 to 72% *(global),* 4 to 72% *(convenience)*, and 9 to 49% *(effectiveness)*. Starting with all least favorable characteristics, and only varying training/infusion characteristics to most favorable levels, predicted probabilities of achieving the best tertiles increased to: 59% (*side effects),* 52% *(convenience)*, 27% *(effectiveness)*, and 26% *(global).* With only favorable SCIG experience, but least favorable training/infusion and other non-infusion characteristics, these probabilities were: 34% (*side effects*), 28% (*effectiveness*), 16% (*global*), and 5% (*convenience*).

For PROMIS Fatigue, predicted impact of attaining the best tertile ranged from 18% with least favorable training/infusion characteristics to 44% with most favorable infusion/training characteristics, and this accounted for all the predicted variation in PROMIS Fatigue. With only favorable SCIG experience, the probability remained at 18%. Finally, for GHP, the predicted probability of attaining the best tertile ranged from 1% with all predictors at least favorable, to as much as 92% with all predictors at most favorable. Thus, the model accounted for almost all the variation in subject self-perceived general health. Appendix Table 8 provides the goodness of fit calibration statistics as well as the receiver operating characteristic (ROC) area under the curves for discriminatory power of each model.

## Discussion

In this analysis of US respondents within a nationally distributed IDF survey, we examined a range of PROs focusing on the direct and indirect impact of treatment-related characteristics. Specifically, we evaluated the importance of SCIG self-infusion efficiency, training, and patient perception of training in the larger context of the chronic nature of PIs – where multiple physician visits, potential infection-related episodes, and associated stress can lead to poor overall perceived health. Conversely, with consistent disease control once IgG therapy has been in place, patients may be expected to maintain improved and stable perceived health [12, 17]. Thus, unsurprisingly, our findings revealed that longer experience with IgG therapy in general was consistently associated with improvement in overall QOL (as measured by GHP).

However, unique to our study is the finding that longer experience with SCIG, specifically > 2 years, was associated with significant improvement in the TSQM *convenience* domain. As IgG therapy requires individualization, the process from SCIG initiation to a fully independent and optimized self-infusion regimen can take time. Our results suggest > 2 years can be required for patients to achieve highest levels of treatment satisfaction, and may reflect the time needed by some patients to determine the right combination of ancillary supplies and infusion parameters (number of sites, volume per site, infusion rate and frequency etc.). Our analysis predicted that efficiency in infusions and training can substantially enhance treatment satisfaction and may lower fatigue beyond what is achievable with SCIG experience alone. Approaches to accelerate optimization of patient satisfaction with improvement in infusion/training characteristics would likely be beneficial to patients and may reduce the number of patients who opt to revert to another treatment solely due to challenges optimizing SCIG rather than clinical need.

Our results indicate training quality, trainer ability, and patient confidence are key factors when considering methods to improve patient outcomes. Ensuring training is easily accessible, and that there are no barriers to training, can also improve treatment satisfaction, observed particularly for TSQM *effectiveness* and *convenience* domains. Respondents who required ≤3 training sessions were associated with higher scores for TSQM *effectiveness* and *side effects*. In addition, total SCIG training lasting < 4 h was associated with a high ease of training. Our results imply that while thorough training with a skilled professional would facilitate greater confidence in SCIG use, the overall duration of training should ideally be concise to ensure the benefits outweigh the burden of time-consuming training. The exact amount of SCIG training, training location, and ease of training will differ at a patient level, but it appears from our findings that it is important to ensure that patients feel confident by their final training session. For example, our results suggest training was perceived to be both easier and more efficient when conducted in the hospital or physician office, and along with greater perceived confidence in turn contributed to better treatment satisfaction; the implication for practice being that this goal may be achieved either via having more patients trained in the hospital or office setting, and/or by implementation of best practices in other training locations.

Further, perceived inconvenience and concerns about needle sticks and about adverse effects have previously been reported to potentially hinder successful SCIG adoption [13]; yet evidence from several clinical studies suggests that patients who do transition to SCIG report, in fact, fewer side effects and better treatment satisfaction than while on IVIG [9–11, 13]. Accordingly, preparing and educating trainers to appropriately educate patients about misplaced concerns and build confidence would be well invested.

Shorter infusion preparation times (specifically < 20 min) were associated with higher TSQM scores for *side effects*, *convenience,* and *global,* thus appearing to offset the unfavorable impact of < 2 years treatment experience. Shorter actual infusion times were associated with higher scores for *side effects* and lower fatigue. Additionally, higher GHP was predicted by fewer infusion sites. These results indicate the importance of having the right ancillary supplies and optimizing infusion set-up. Patient education, as part of SCIG training, should include information on needle length/gauge, number of sites and rotation, and different flow rate tubing sets. Finally, higher concentration SCIG products were associated with shorter infusion times. Understanding these variables and using the right supplies could lead to reduced infusion times, which our results reveal led to higher levels of satisfaction.

Our results also suggested that the site of training, especially the hospital/doctor’s office, was associated with more efficient infusions and training time, suggesting that ensuring patients are well trained *prior to* transitioning to home-based self-infusion is potentially beneficial. In turn, we found that favorable training and efficient infusions can substantially increase the probability of patients experiencing higher treatment satisfaction and QOL, independent of characteristics such as age, time on treatment, or SCIG experience – this was apparent across all TSQM domains.

The data presented here builds upon trends reported from a previous IDF survey [18], which highlighted issues of fatigue and the importance of developing individual patient treatment plans. The importance of treatment satisfaction has been previously discussed in a literature review by Barbosa et al. where greater treatment satisfaction was associated with better compliance and improved persistence across a range of disease areas, including chronic diseases [19]. The review additionally supported the notion that less complex treatments can also improve treatment satisfaction and regimen adherence. Thus, applied to IgG infusions, expected innovations that simplify the SCIG procedure for patients, for example, pre-filled syringes or infusion wearables, are anticipated to contribute to reducing treatment complexity by eliminating steps and supplies required for infusions. Evidence in asthma suggested that the use of inhaler devices was associated with better patient satisfaction and compliance [20]. Patient education on anticipated IgG therapy innovations, to help patients with PI understand their disease and treatment options, may be highly beneficial for enhancing satisfaction and compliance in IgG therapy, and also improve clinical and economic outcomes [21].

We acknowledge some limitations are inherent with patient-reported surveys. Although this study was conducted using the largest US database of patients with PI, as a non-probability sample, generalization of results to the larger population should be made with caution. As with most patient surveys, responses could not be independently verified with patients’ physicians. The direction of causation is not always unambiguous – the observed association between the TSQM *side effects* domain or fatigue and efficient infusions, in particular, may reflect that subjects with fewer perceived side effects or less fatigue were able to complete infusions more efficiently, rather than vice versa. If so, this would nevertheless suggest that innovative advances in infusion methods may enable even those with some side effects or fatigue to infuse more efficiently. Lastly, we considered the potential impact of incorporating parent/caregiver proxy responses rather than excluding pediatric patients. However, no significant differences were observed when pediatric responses (*n* = 40) were removed from the analysis; therefore, the impact of using a combined population was considered negligible. Despite potential limitations, our model predicted a dramatic impact on patient QOL of varying identified predictors and highlights some key areas where simple improvements could make crucial differences to patients.

## Conclusions

Respondents with favorable SCIG training and efficient infusions had significantly higher GHP and TSQM scores, particularly *convenience*, *effectiveness,* and *side effects*, compared with those with a poorer training experience and longer infusions. Increased experience, especially over 2 years, with SCIG treatment was consistently associated with higher PROs, but our findings also show that better training and infusion characteristics were associated with higher patient treatment satisfaction beyond what was achieved with accumulation of SCIG experience. We propose that improvements in SCIG training, to ensure patient confidence at training completion, and methods to minimize infusion preparation/duration could result in improved GHP and treatment satisfaction. Enhancements in SCIG therapy that reduce the burden of treatment and improve patient convenience have the potential to play an important role in accelerating this patient learning curve and achievement of high treatment satisfaction toward the ultimate goal of improving health outcomes.

## Methods

### Survey population, design, and administration

Using the IDF patient database, 11,232 US-based patients with PI, or their parent/caregiver if < 18 years old, were contacted online regarding an unincentivized online survey. The survey contained 74 questions, including PROs — (i) the TSQM, (ii) the PROMIS Fatigue Adult Short Form (SF)7a or Parent/Caregiver Proxy SF10, and (iii) GHP, to measure overall health-related QOL. Experience with IgG infusion characteristics and training characteristics were assessed. Patient responses, on an anchored 1–7 scale (where 1 = poor experience/perception; 7 = excellent experience/perception), were used to assess the quality of respondents’ SCIG training and infusion characteristics. Infusion and training efficiency were assessed in terms of duration and frequency of the sessions. Full details of IDF surveys can be found at the following link: (https://primaryimmune.org/sites/default/files/2017-Patient-Reported-Outcomes-and-Treatment-Survey.pdf).

### Conceptual framework: infusion and training characteristics to PROs

**Fig. 3:**
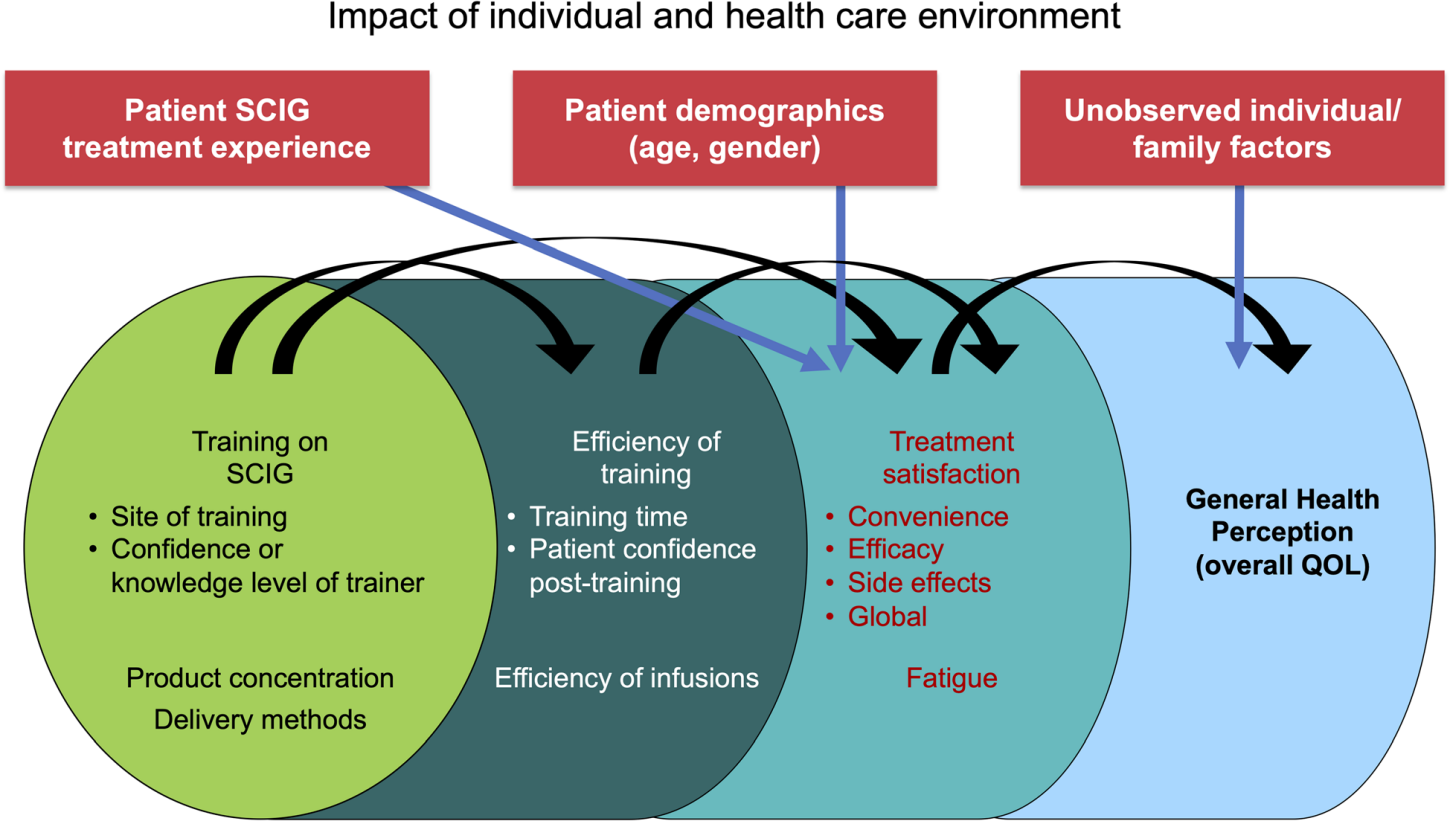
A continuum of health outcomes: an adaptation to subcutaneous immunoglobulin self-infusions in primary immune deficiency. Conceptual framework adapted from Wilson IB, et al. *JAMA*. 1995;273(1):59–65. QOL, quality of life; SCIG, subcutaneous immunoglobulin

We adapted the Wilson-Cleary conceptual framework [22] of a continuum of health outcomes to explicitly incorporate the impact of IgG infusion and training characteristics on training/infusion efficiency, and in turn on patient-reported treatment satisfaction, fatigue, and perceived health [12] (Fig. 3).

In this framework, GHP represents the culminating target outcome as an individual's integrated well-being consisting of biological, psychological, environmental, and social aspects [12]*.* Immediately preceding on our conceptualized continuum (Fig. 3), treatment satisfaction [23] and fatigue [24] influenced perceived health, and were in turn driven by training/infusion characteristics. We simultaneously modeled the role of disease/treatment history and respondent demographics.

### TSQM

Treatment satisfaction was assessed using the TSQM, which measures patients’ satisfaction with medication [25], with modified instructions asking patients to refer to their IgG infusion when responding on medication. Satisfaction is reported in terms of the total score and four domain scores: *effectiveness*, *side effects*, *convenience*, and *global.* Following the TSQM scoring algorithm, raw scores were transformed to a 0–100 scale (where 0 = poorest satisfaction; 100 = perfect satisfaction) [25].

### PROMIS Fatigue-SF7a/10

Fatigue was assessed using the PROMIS Fatigue SF7a/SF10 for adult and parent/caregiver responses, respectively [26]. Summed scores were transformed to PROMIS Fatigue T-scores using previously published concordance tables from a large National Institutes of Health-funded initiative [26, 27]. The Fatigue SF7a (containing 7 items) was chosen to permit a representation of fatigue levels ranging from the item “how often did you experience extreme exhaustion” (where an average response would represent a relatively high level of fatigue) compared with “how often did you have enough energy to exercise strenuously” (where an average response would indicate relatively less fatigue). Finally, use of the PROMIS Fatigue T-score allowed comparison to a population norm (mean score of 50 and standard deviation [SD] of 10) [28].

### Training and infusion characteristics

Favorable infusion and training characteristics were defined as follows:

- *Favorable training:* (i) efficiency of training, (ii) the respondent’s self-perception of their trainer (high [6, 7] scores for competence and knowledge of trainer, and satisfaction with quality of training), and (iii) absence of training barriers. Efficient training characteristics included: requiring ≤3 training sessions; a total SCIG training time ≤4 h; individual training sessions ≤2 h; ease of training rated 6–7; and confidence after training rated 6–7.
- *Efficient infusions:* defined as reported actual infusion time of ≤2 h; infusion preparation duration of ≤20 min; and total infusion duration (including preparation and clean-up) of ≤3 h, or lower frequency of infusions.

### Analysis population

Respondents who indicated they were not currently receiving IgG subcutaneously were removed from the analysis. Incomplete surveys or those with incongruent responses (i.e. those with incompatible responses such as selecting currently receiving SCIG, but citing an IVIG product) were removed.

### Univariate statistical analysis

Associations between infusion, training characteristics, and each PRO were first examined individually. PRO scores were classified by tertiles (best, intermediate, and worst) based on ranking respondent scores. Ties at tertile thresholds were resolved by placing respondents into one or other category to even the number of patients in each tertile as much as possible. The best tertile was targeted as the desired outcome. All other variables were either originally measured in categories, or categorically classified, for the purposes of analysis. All categorical inferences were based on the Chi-square test.

### Multiple logistic and linear regressions

Multivariate logistic models were estimated to identify predictors of the desired best tertile for GHP, TSQM, and PROMIS Fatigue T-scores. Stata (version 15.1) was used for all analyses. Independent predictors were identified by the backwards elimination method. The threshold for being retained in the model was a multivariate *p*-value of < 0.1.

Outcomes and covariates were also evaluated on a continuous scale using linear regression, where appropriate. SCIG training and infusion times were found to have positively skewed distributions, therefore were analyzed on the log scale. The predictive ability of the continuous models was evaluated by the R^2^ statistic.

The discrimination ability of the logistic regression models was examined using ROC curves [29]. The calibration of the models was examined using the Hosmer-Lemeshow goodness of fit test [30].

### Correlation analyses

Correlation analyses were used to examine the strength of association between training variables such as training location, knowledge of trainer, ease of training and total SCIG training time. Three of the variables (knowledge of trainer, ease of training, and total SCIG training time) were measured on either an ordinal (rank ordered) or continuous scale, and the association between each pair of these variables was assessed using Spearman’s rank correlation. The remaining variable, training location, was categorical in nature; therefore a Kruskal-Wallis test was used to compare the training location to other variables.

### Predicted probabilities of desirable PROs

The estimated multiple logistic regressions were used to generate predicted probabilities (by exponentiating the estimated logistic link function [31]) of achieving the best PRO tertiles at (i) least and most favorable levels of all characteristics, (ii) most favorable levels of training/infusion characteristics, but least favorable levels for all non-infusion characteristics, and (iii) most favorable levels for SCIG experience, but least favorable levels for training/infusion and other non-infusion characteristics.

## Data Availability

CSL will only consider requests to share survey data that are received from systematic review groups or bona-fide researchers.

## Appendix

**Table 8:** Examination of PRO model diagnostics

Type of test of models	Model discrimination (ROC curve)		Model calibration – Hosmer-Lemeshow test
Characteristics	AUC	95% CI	***p***-value
**General health perception**			
	0.84	0.80, 0.89	0.78
**TSQM scores**			
Total	0.66	0.60, 0.72	0.74
*Effectiveness*	0.61	0.56, 0.67	0.92
*Side effects*	0.69	0.64, 0.75	0.49
*Convenience*	0.69	0.63, 0.76	0.81
*Global*	0.69	0.63, 0.74	0.19
**PROMIS Fatigue**			
	0.62	0.56, 0.68	0.29

The AUC and 95% CI summarize the discriminatory ability of the relevant PRO model. The Hosmer-Leweshow *p*-value summarizes the goodness of fit test under the null hypothesis of lack of fit – all values *p* > 0.05 suggest good calibration of the model to the data

*AUC* area under the curve, *CI* confidence interval, *PRO* patient-reported outcome, *PROMIS* Patient-Reported Outcome Management Information System, *ROC* receiver operating characteristic, *TSQM* Treatment Satisfaction Questionnaire for Medication

## Abbreviations

CI: Confidence interval
CC: Correlation coefficient
GHP: General health perception
IDF: Immune Deficiency Foundation
IgG: Immunoglobulin G
IQR: Interquartile range
IVIG: Intravenous immunoglobulin
OR: Odds ratio
PI: Primary immunodeficiency
PRO: Patient-reported outcome
PROMIS: Patient-Reported Outcomes Management Information System
PROMIS SF7a/SF10: Short form 7a (adult)/short form 10 (parent/caregiver proxy)
QOL: Quality of life
ROC: Receiver operating characteristic
SCIG: Subcutaneous immunoglobulin
SD: Standard deviation
TSQM: Treatment Satisfaction Questionnaire for Medication
US: United States

## References

[1] Jiang F, Torgerson TR, Ayars AG (2015). Health-related quality of life in patients with primary immunodeficiency disease. Allergy, Asthma Clin Immunol.

[2] Picard C, Bobby Gaspar H, Al-Herz W (2018). International Union of Immunological Societies: 2017 primary immunodeficiency diseases committee report on inborn errors of immunity. J Clin Immunol.

[3] Amaya-Uribe L, Rojas M, Azizi G (2019). Primary immunodeficiency and autoimmunity: a comprehensive review. J Autoimmun.

[4] Boyle JM, Buckley RH (2007). Population prevalence of diagnosed primary immunodeficiency diseases in the United States. J Clin Immunol.

[5] Kafal AR, Vinh DC, Langelier MJ (2018). Prefilled syringes for immunoglobulin G (IgG) replacement therapy: clinical experience from other disease settings. Expert Opin Drug Deliv.

[6] Vermeire S, D'Heygere F, Nakad A (2018). Preference for a prefilled syringe or an auto-injection device for delivering golimumab in patients with moderate-to-severe ulcerative colitis: a randomized crossover study. Patient Prefer Adherence.

[7] Megari K (2013). Quality of life in chronic disease patients. Health Psychol Res.

[8] Burton J, Murphy E, Riley P (2010). Primary immunodeficiency disease: a model for case management of chronic diseases. Prof Case Manag.

[9] Nicolay U, Kiessling P, Berger M (2006). Health-related quality of life and treatment satisfaction in north American patients with primary immunedeficiency diseases receiving subcutaneous IgG self-infusions at home. J Clin Immunol.

[10] Espanol T, Prevot J, Drabwell J (2014). Improving current immunoglobulin therapy for patients with primary immunodeficiency: quality of life and views on treatment. Patient Prefer Adherence.

[11] Berger M (2008). Subcutaneous administration of IgG. Immunol Allergy Clin N Am.

[12] Seeborg FO, Seay R, Boyle M (2015). Perceived health in patients with primary immune deficiency. J Clin Immunol.

[13] Berger M, Murphy E, Riley P (2010). Improved quality of life, immunoglobulin G levels, and infection rates in patients with primary immunodeficiency diseases during self-treatment with subcutaneous immunoglobulin G. South Med J.

[14] Koterba AP, Stein MR (2015). Initiation of immunoglobulin therapy by subcutaneous administration in immunodeficiency patients naive to replacement therapy. Allergy, Asthma Clin Immunol.

[15] Abolhassani H, Sadaghiani MS, Aghamohammadi A (2012). Home-based subcutaneous immunoglobulin versus hospital-based intravenous immunoglobulin in treatment of primary antibody deficiencies: systematic review and meta analysis. J Clin Immunol.

[16] Jolles S, Stein MR, Longhurst HJ (2011). New Frontiers in subcutaneous immunoglobulin treatment. Biol Ther.

[17] Gardulf A, Bjorvell H, Gustafson R (1993). The life situations of patients with primary antibody deficiency untreated or treated with subcutaneous gammaglobulin infusions. Clin Exp Immunol.

[18] Rider NL, Kutac C, Hajjar J (2017). Health-related quality of life in adult patients with common variable immunodeficiency disorders and impact of treatment. J Clin Immunol.

[19] Barbosa CD, Balp MM, Kulich K (2012). A literature review to explore the link between treatment satisfaction and adherence, compliance, and persistence. Patient Prefer Adherence.

[20] Small M, Anderson P, Vickers A (2011). Importance of inhaler-device satisfaction in asthma treatment: real-world observations of physician-observed compliance and clinical/patient-reported outcomes. Adv Ther.

[21] Balkrishnan R (2005). The importance of medication adherence in improving chronic-disease related outcomes: what we know and what we need to further know. Med Care.

[22] Wilson IB, Cleary PD (1995). Linking clinical variables with health-related quality of life. A conceptual model of patient outcomes. JAMA..

[23] Westaway MS, Rheeder P, Van Zyl DG (2003). Interpersonal and organizational dimensions of patient satisfaction: the moderating effects of health status. Int J Qual Health Care.

[24] Cook KF, Bamer AM, Roddey TS (2012). A PROMIS fatigue short form for use by individuals who have multiple sclerosis. Qual Life Res.

[25] Atkinson MJ, Sinha A, Hass SL (2004). Validation of a general measure of treatment satisfaction, the treatment satisfaction questionnaire for medication (TSQM), using a national panel study of chronic disease. Health Qual Life Outcomes.

[26] Health Measures. Patient reported outcome measurement information system (PROMIS) guide 2019 [Available from: http://www.healthmeasures.net/].

[27] Rose M, Bjorner JB, Gandek B (2014). The PROMIS physical function item bank was calibrated to a standardized metric and shown to improve measurement efficiency. J Clin Epidemiol.

[28] Rothrock NE, Hays RD, Spritzer K (2010). Relative to the general US population, chronic diseases are associated with poorer health-related quality of life as measured by the patient-reported outcomes measurement information system (PROMIS). J Clin Epidemiol.

[29] Steyerberg EW, Vickers AJ, Cook NR (2010). Assessing the performance of prediction models: a framework for traditional and novel measures. Epidemiology..

[30] Hosmer DW, Lemeshow S, Sturidvant RX (2013). Applied logistic regression.

[31] SAS/STAT(R) 9.2 User's Guide, Second Edition 2019.

